# Validating peer-led assessments of CPR performance

**DOI:** 10.1016/j.resplu.2020.100022

**Published:** 2020-08-06

**Authors:** Anna Abelsson, Carl Gwinnutt, Paul Greig, Jonathan Smart, Kevin Mackie

**Affiliations:** a Jönköping University, School of Health Sciences, PO Box 1026, 551 11, Jönköping, Sweden; b Resuscitation Council (UK), Tavistock Square, London, WC1H 9HR, UK; c Department of Anaesthetics, Guy’s and St Thomas’s NHS Foundation Trust, Westminster Bridge Road, London, SE1 7EH, UK; d Academic Visitor, Nuffield Department of Clinical Neurosciences, University of Oxford, West Wing Level 6, John Radcliffe Hospital, Oxford, OX3 9DU, UK; e Innosonian Europe, Farnborough, Hampshire, GU14 8FF, UK

**Keywords:** Assessment, Cardiopulmonary resuscitation, Manikin

## Abstract

**Background:**

A patient’s survival from cardiac arrest is improved if they receive good quality chest compressions as soon as possible. During cardiopulmonary resuscitation (CPR) training subjective assessments of chest compression quality is still common. Recently manikins allowing objective assessment have demonstrated a degree of variance with Instructor assessment. The aim of this study was to compare peer-led subjective assessment of chest compressions in three groups of participants with objective data from a manikin.

**Method:**

This was a quantitative multi-center study using data from simulated CPR scenarios. Seventy-eight Instructors were recruited, from different backgrounds; lay persons, hospital staff and emergency services personnel. Each group consisted of 13 pairs and all performed 2 ​min of chest compressions contemporaneously by peers and manikin (Brayden PRO®). The primary hypothesis was subjective and objective assessment methods would produce different test outcomes.

**Results:**

13,227 chest compressions were assessed. The overall median score given by the manikin was 88.5% (interquartile range 71.75–95), versus 92% (interquartile range 86.75–98) by observers. There was poor correlation in scores between assessment methods (Kappa −0.051 – +0.07). Individual assessment of components within the manikin scores demonstrated good internal consistency (alpha ​= ​0.789) compared to observer scores (alpha ​= ​0.011).

**Conclusion:**

Observers from all backgrounds were consistently more generous in their assessment when compared to the manikin. Chest compressions quality influences outcome following cardiac arrest, the findings of this study support increased use of objective assessment at the earliest opportunity, irrespective of background.

## Introduction

Sudden cardiac arrest is one of the leading causes of death in Europe, estimated to affect between 0.5 and 1.0 per 1000 of the population, or 350,000–700,000 persons each year.^1^, ^2^, ^3^ With less than 10% of patients surviving, there is enormous scope to improve outcomes.^4^ One of the key mechanisms by which improvement can be achieved is by prompt and effective delivery of good-quality chest compressions (CC), as defined by compression rate,^5^^,^^6^ incomplete release,^7^^,^^8^ hand position^9^^,^^10^ and depth.^11^, ^12^, ^13^ Futhermore several studies have shown CPR quality to be a critical determinant of survival after cardiac arrest.^15^, ^16^, ^17^

CPR training is delivered using manikins, with an assessment of competence made by Instructor observation. Subjective assessment has been shown to be inconsistent when compared with objective assessment, but has not been complared amongst laypersons, healthcare professions and emergency care personnel.^18^, ^19^, ^20^, ^21^ The primary objective of the present study was to investigate if there was any difference between contemporaneous observer and manikin assessment of CC skills amongst three groups of Instructors from differing back grounds; laypersons (French Red Cross volunteers), Dutch hospital healthcare professionals (HCPs) and Swedish Emergency Service personnel (Firefighters) using an assessment based on the European Resuscitation Council 2015 Guidelines.

## Method

The aim of this study was to compare peer-led subjective assessment of chest compressions in three groups of Instructors from different backgrounds with contemporaneous objective data from a manikin. Instructors were chosen as they are familiar with carrying assessment of CCs. The primary hypothesis was that subjective (Instructor) and objective (manikin) assessments would yield different results within in all groups of participants. Secondary hypotheses were that there would be no agreement in the pass marks between the observers and the manikin for the assessments for any of the groups when tested over a range of arbitrary pass marks’, and we would find poor internal consistency across the component skills of CCs.

### Participants

Seventy-eight Instructors from differing backgrounds and three European countries participated as 3 equal groups of 26, each of which was then divided into 13 pairs. The three groups consisted of:

Group 1. 8 females, 18 males, lay members of the French Red Cross.
Group 2. 10 females, 16 males.,Dutch hospital-based healthcare professionals.
Group 3. 4 females, 22 males, Swedish Emergency Service personnel.

### Data collection

One of each pair was asked to perform CCs for 2 ​min. During this time, both the manikin (Brayden PRO® Manikin, Innosonian, Europe) and the non-performing Instructor of the pair were simultaneously recording the participants’ performance. At the end of the 2 ​min, there was a short break and the roles of participant and observer were reversed and a further 2 ​min of CCs performed. For each 2-min session, both the manikin and Instructors produced:

• a score of each participant’s performance of CC rate, depth, hand position and complete release over the 2 ​min period
• an overall score for each participant (a ‘pass mark’)

The overall scores for each individual were given as a separate, subjective score by the Instructors based upon their assessment of the individual skills and the manikin score was calculated from the scores given to each individual component.

All assessments were made with the manikin placed on a hard surface. The individual participants did not receive any feedback about their performance during their assessment. The manikin required 35–45 ​kg force to achieve a chest compression depth of 5–6 ​cm and was calibrated during development, using expert opinion to give different weightings to the components being assessed for candidates of differing experience. To allow a direct comparison, observer scores were weighted to the asame degree.

For each group, the assessor used a standardised score sheet including skills of CC rate, depth, hand position and complete release. All skills were scored individually by both observers and manikin using weighted CC metrics according to the participant group. An overall score based on each component was made and converted to a percentage to allow comparisons (the ‘pass mark’) set at 5% intervals from 50 to 90%.

### Data analysis

A descriptive and inferential analysis was conducted using the IBM Statistical Package for the Social Sciences (SPSS) 24.0. Data were not normally distributed according to the Shapiro-Wilk test, so non-parametric methods were used for comparisons. Comparisons between the three professional groups were assessed using an independent samples Kruskall-Wallis test, and pairwise comparisons were made using a Bonferroni correction for multiple tests. Comparison of the scores yielded by each assessment method were compared using a related-samples Wilcoxon signed-rank test and Spearman’s rho. Individual components of CCs of compression rate, compression depth, complete release and correct hand position within the both assessments were tested for internal consistency using Cronbach’s alpha. Correlation between the assessment scores were tested using Spearman’s rho. Tests of agreement were conducted for dichotomised pass-fail outcomes yielded by the manikin and observer assessments. Pass marks were arbitrarily set in 5% increments from 50 to 90%. Receiver-operating characteristic (ROC) curves were plotted to test the ability of the observers to correctly classify participants, using the manikin assessment outcome as the ‘gold standard’ and Cohen’s Kappa values calculated at each step.

## Ethical considerations

The study conformed with the ethical principles according to The Declaration of Helsinki, Medical Research Involving Human studies^22^ and local requirements for volunteer studies of this type. In none of the countries was ethical approval deemed necessary. None of the participants were vulnerable or in a dependent position, all received information about the study prior to obtaining informed consent and all were given the right to withdraw at any point. Privacy and confidentiality were maintained throughout the study and all data was anonymised.

## Results

### Comparison of assessment methods

**Fig. 1:**
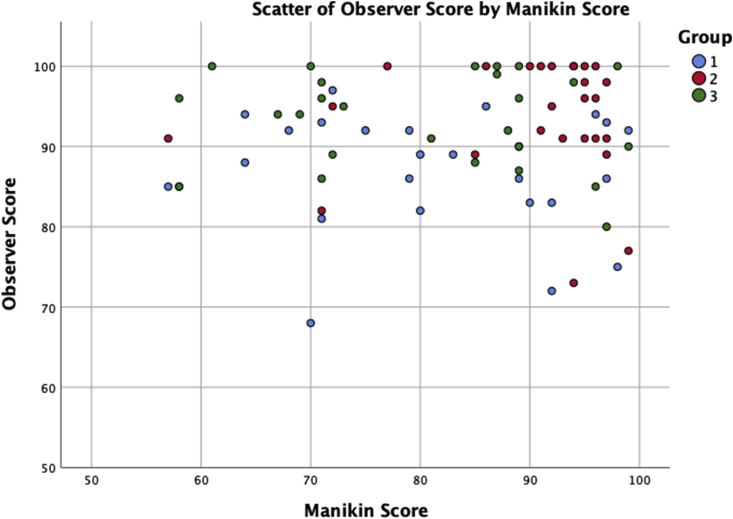
Scatterplot of manikin versus observer scores.

The 78 participants performed a total of 13,227 chest compressions. The overall median score (%) for individual performance given by the manikin was 88.5 (interquartile range (IQR) 72–95), versus 92 (IQR 87–98) by observers. Scores yielded by each assessment method differed significantly from one another. Across all groups the median score given by the manikin was significantly lower than that from observers (p ​< ​0.001) (Table 1). The scores given by each assessment method also correlated very poorly. No significant correlation could be detected overall (Spearman’s rho ​= ​0.039, p ​= ​0.734), or at the individual group levels (1: rho ​= ​−0.034, p ​= ​0.869, 2: rho ​= ​−0.121, p ​= ​0.556; 3: rho ​= ​−0.1, p ​= ​0.627) (Figure 1).

**Table 1:** Overall scores (%) median interquartile range (IQR) for manikin and observers. All values rounded to nearest integer.

Group	Manikin Score (%) Median (IQR)	Observer Score (%) Median (IQR)
1	80 (71–92)	89 (83–92)
2	94 (89–96)	96 (91–100)
3	85 (71–89)	95 (89–99)

Sequential pass marks for individuals, from 50% to 90%, were tested for agreement. Below 70%, no candidates were failed by the observers. Between 70% and 90%, Kappa ranged from −0.024 to +0.07. The Area Under the Curve (AUC) the Receiver Operating Characteristics (ROC) curves show that the observer assessment performs little better than chance in predicting the outcome of manikin assessment (Table 2).

**Table 2:** Pass marks 70–90% and number of participants, n (%) who achieved the score by manikin and observer assessment.

Pass mark (%)	Participants achieving pass mark by manikin n (%)	Participants achieving pass mark by observer n (%)	Kappa	ROC AUC
70	67 (85.9)	77 (98.7)	−0.024	0.546
75	55 (70.5)	75 (96.2)	0.01	0.547
80	51 (65.4)	73 (94.8)	−0.051	0.537
85	47 (60.2)	67 (85.9)	−0.022	0.58
90	33 (42.3)	51 (65.4)	0.07	0.524

Individual scores for compression rate, compression depth, hand position and complete release assessed by the manikin showed good internal consistency (Cronbach’s alpha ​= ​0.789), although this might be predicted given that the overall score is calculated algorithmically by the manikin. Conversely, the individual assessment components within the observer scores demonstrated very poor internal consistency (Cronbach’s alpha ​= ​0.011). The assessment of hand position was particularly unreliable; were this item to be deleted from both the manikin and observer assessment, alpha increased to 0.811 and 0.344 respectively.

### Comparison of professional groups

Table 3 demonstrates that the manikin assessed group 2 significantly more highly than both groups 1 and 3 (p ​= ​0.022, p ​= ​0.018), but did not detect a difference between groups 1 and 3 (p ​= ​1.0). The observers however rated both groups 2 and 3 significantly more highly than group 1 (p – 0.001, p ​= ​0.007), but did not detect a significant difference between group 2 and 3 (p ​= ​0.007). Comparisons of the individual assessment components are presented in Table 4.

**Table 3:** Pairwise comparisons of scores for groups by manikin and observers. Median, Interquartile Range (IQR).

Comparison	Manikin Median, (IQR)	Observer Median (IQR)
Group 2 v 1	94 (89–96) v 80 (71–92) p ​= ​0.022	95.5 (91–100) v 88.5 (83–92) p ​= ​0.001
Group 2 v 3	94 (89–96) v 85 (71–89) p ​= ​0.018	95.5 (91–100) v 94.5 (89–99) p ​= ​1.0
Group 1 v 3	80 (71–92) v 85 (71–89) p ​= ​1.0	88.5 (83–92) v 94.5 (89–99) p ​= ​0.007

**Table 4:** Comparison of performance metrics by group as assessed by the manikin. All values rounded to nearest integer. ∗Pairwise comparisons include a Bonferroni correction for multiple comparisons.

	Group 1	Group 2	Group 3	p values	
	Median % (IQR)	Median % (IQR)	Median % (IQR)	Overall	Pairwise∗
Overall compression rate min^−1^	118 (112–124)	102 (97–107)	116 (106–122)	<0.001	2-3: 0.001 2-1: <0.001 3-1: 0.860
<100 min^−1^	4 (4–5)	9 (4–38)	0 (0–3)	<0.001	2-3: <0.001 2-1: 0.06 3-1: 0.006
100-120 min^−1^	26 (2–80)	80 (38–92)	45 (4–96)	0.072	–
>120 min^−1^	70 (13–94)	0 (0–2)	14 (0–71)	<0.001	2-3: 0.010 2-1: <0.001 3-1: 0.409
Overall compression depth (cm)	6 (5–6)	6 (5–6)	6 (6-6)	0.006	2-3: 0.006 2-1: 1.0 3-1: 0.073
<5 ​cm	0 (0–2)	0 (0–13)	0 (0-0)	0.006	2-3: 0.007 2-1: 1.0 3-1: 0.049
5–6 ​cm	89 (37–97)	73 (57–93)	63 (4–96)	0.392	–
>6 ​cm	6 (0–59)	3 (0–30)	38 (4–96)	0.013	2-3: 0.013 2-1: 1.0 3-1: 0.0111
Correct hand position	100 (100-100)	100 (100-100)	100 (99–100)	0.668	–

## Discussion

Currently, the main method of CPR training is using manikins combined with an assessment of competence in the skills of CC and rescue breathing conducted by instructors trained to observe performance. Widespread use of objective assesment still remains low amongst Resuscitation Council UK Instructors. In a recent survey, (40% response rate, 138/342 course centres) the sole use of objective assessment of chest compression skills courses ranged from 12% for hand position to 21% for CC rate and 14% to 25% 14% on BLS and ALS courses respectively. respectively (M. Gwinnutt, personal communication).

We believe this is the first study to compare peer assessment of chest compressions by experienced Instructors with those made by a manikin (Brayden PRO® Manikin, Innosonian, Europe), recruiting participants from three very different backgrounds and experience of cardiac arrest management. We found that Instructors consistently overestimate the quality of CCs when assessed as individual skills and in the identification of those who have achieved a predetermined ‘pass mark’.

There have been several previous studies of the divergence between objective and subjective assessments. Lynch et at compared experienced CPR Instructors assessment with a skill reporting manikin and found that Instructors assessed inadequate depth and incorrect hand position as correct 55% and 49% of the time respectively.^18^ Sanchez et al. evaluated the assessment of correct external CCs evaluated by three expert instructors and a manikin.^19^ They reported that they found the degree of agreement and uniformity among the 3 evaluators and manikin to be poor, with a high degree of dispersion with no defined trend. Brenner et al. analyzed data collected during simulation training sessions for residents, medical students, and nursing students and similarly found that Instructor assessment of chest compression rate, depth, and fraction demonstrated poor sensitivity and specificity when compared to the data from a simulation manikin.^20^ González et al. found subjective assessment often very inaccurate and has called into question the reliability and validity of this approach.^21^ More recently Hansen et al. compared BLS Instructors versus a manikin to evaluate CC skills of medical students. In 90 assessments performed by 16 instructor pairs, they identified correct CCS with a sensitivity of 0.96 [95% confidence interval] (CI) ​= ​0.79–1) and a specificity of 0.05 (95% CI ​= ​0.01–0.14). Instructors passed 90% of students compared to 2% by the manikin.^21^ Our findings are in broad agreement with these studies. However, all were only looking at a single specialty groups of participants. The analysis of the performance of over 13,000 ​CCs by 76 participants from three different backgrounds clearly supports our primary hypothesis that the two assessment methods yield different outcomes. Subjective assessment was consistently more generous than the objective assessments measured by the manikin. All analyses of data pointed in the same direction, confirming significant differences between the performance of the manikin and observers with no correlation.

Our data also support the hypothesis that the test methods yield very poor agreement on test outcome across a range of potential pass marks. Given that observers’ assessments perform little better than chance in predicting manikin outcomes, these finding may have implications for those teaching CPR.

When comparisons were made between professional groups, these showed that the manikin scored participants in group 2 higher than those in groups 1 and 3. This may be a reflection that the participants in group 2 were all in-hospital healthcare professionals, exposed to and managing cardiac arrests far more frequently than those participants in groups 1 and 3. In addition they may, by virtue of their profession, have received more frequent training and assessment in CPR and be aware of the need for good quality CPR and its effect on outcomes than the other two groups.^5^^,^^6^^,^^13^^,^^15^^,^^16^ One might also speculate that the observer’s expectations of the performance of group 2 participants, being peers of those performing, may have subconsciously biased their assessment thereby leading to higher scores.

For compression rate the median performance of all groups was within the target range of 100–120 min^−1^, with group 2 having a significantly slower rate than groups 1 and 3. This again may reflect the greater experience of group 2 participants and an understanding of the effect of excess rates of CC on their efficacy. Whilst the median performance of depth of CCs for all groups was within the target of 5–6 ​cm, Group 3 performed deeper chest compressions with 55% assessed as too deep. A possible explanation for the excessive depth amongst group 3 may be that the American Heart Association (AHA) Guidelines 2010 were that depth of compressions should be at least 5 ​cm.^23^ As a result, some manikins were designed to record all compressions >6 ​cm as 100% correct and so this may have influenced performance. In order to achieve ‘satisfactory completion’ of an assessment of CCs, participants may be over-compressing in the knowledge that previously they would not be penalised. Recognising the reduced efficacy, and potential for internal injury with excessive compression depths^24^ the current AHA guidelines now recommend that during manual CPR, rescuers should perform chest compressions to a depth of at least 2 inches or 5 ​cm for an average adult, while avoiding excessive chest compression depths (greater than 2.4 inches or 6 ​cm).^25^

Group 3 performed significantly deeper chest compressions than groups 1 and 2 (who did not differ from one another). Whilst the median performance of groups 1 and 2 was within the target of 5–6 ​cm, group 3 was marginally above the upper limit of the target range. This was reflected in the proportional data, which demonstrated that, although this significantly differed only from group 2.

There were very similar findings in assessment of the accuracy of hand position with both assessment methods having median score of 100% for all groups suggesting both methods are unreliable. When this component is removed from the analysis of the data, the internal consistency improves for both observer and manikin assessment. The findings amongst the observers may be a reflection that in the same way complete release is difficult to assess by observation,^26^ the same may be expected of hand position leading to minimal distribution of scores. There are two possible explanations for this in the manikin group, firstly the test item may not be measuring the same underlying educational construct and secondly the manikin may be imprecise in its recognition of hand position.

## Conclusion

Subjective assessment of CC skills is difficult to perform consistently and accurately even for experienced Instructors as this study found. We believe this is the first study to compare experienced participants, from three different backgrounds and potentially varying exposure to cardiac arrests using subjective peer review versus contemporaneous objective assessment by a manikin. The contemporaneous assessment of over 13,000 ​CCs performed by 78 participants are sufficiently different as to be informative, and we found an over-estimation of performance consistently in all three groups during peer-led assessment. As a result, a case can be made for more robust assessment methods, and probably should be based on the objective measures yielded by the use of manikin of the type used in this study which would be expected to be more repeatable, simpler for assessors to interpret, and less prone to biases. This reflects the conclusions of studies which have proposed the need for objective and technologically-supported measures of chest compression quality during resuscitation education to train rescuers to ensure they provide high-quality CCs. The data also suggest that consideration should be given to determining agreed pass marks, particularly for those who have a responsibility for standard-setting. Clearly this will require the input of educationalists and expert clinicians with such experience.

## Limitations

Although we found differences in performance between assessment techniques, it is easy to assume that the poorer performance by group 1 is by virtue of the fact that they are laypersons. However, we did not investigate what may have happened if a lay person assessed a doctor instead of another lay person, and vice versa and whether this would have produced the same pattern of results. Performance during a scenario and using a manikin does not include all the other factors that individuals may face when managing a cardiac arrest and this may have influenced individuals’ performances. On the other hand, it may be that because of the lack of external pressures, some individuals may have performed better than when faced with a victim of cardiac arrest. Several other factors may have influenced our findings; firstly the ‘Hawthorne Effect’, in which individuals modify an aspect of their behaviour in response to their awareness of being observed.^27^ As a result, participants may have performed more competently as a result of being observed by their peers. Secondly, the ‘carry-over’ effect, whereby subsequent performance of tasks may be influenced by previous experiences, and thirdly, the results of the observer scores may have been influenced by the ‘Halo’ effect whereby peer groups may have not wished to show their colleagues as performing badly. Although these effect may have been limited by concurrent videoing of performance, this would not have provided any anonimity.

## Funding

This research received no specific grant from any funding agency in public, commercial or not-for-profit sectors.

## Authors’ contributions

AA, KM and JS collected the data. PG analyzed the data and CG and AA wrote the paper. All authors critically revised and approved the final version of the paper.

## Declaration of competing interest

The authors declare the following financial interests/personal relationships which may be considered as potential competing interests: AA and PG have no conflicts of interest. CG and KM have provided unpaid advice to Innosonian Europe for work in development of the manikin software. JS has a paid position at Innosonian Europe.
